# Physico-Chemical Characteristics and Functional Properties of Chitin and Chitosan Produced by *Mucor circinelloides* Using Yam Bean as Substrate

**DOI:** 10.3390/molecules16087143

**Published:** 2011-08-23

**Authors:** Ana Elizabeth C. Fai, Thayza C.M. Stamford, Thatiana M. Stamford-Arnaud, Petrus D´Amorim Santa-Cruz, Marta C. Freitas da Silva, Galba M. Campos-Takaki, Tânia L.M. Stamford

**Affiliations:** 1 Nutrition Program, Federal University of Pernambuco, Recife 50670-901, Brazil; Email: bethfai@yahoo.com.br; 2 Physiology and Pathology Department, Federal University of Paraiba, João Pessoa 58051-900, Brazil; Email: thayzastamford@yahoo.com.br; 3 Material Science Program, Federal University of Pernambuco, Recife 50670-901, Brazil; Email: thatianaarnaud@hotmail.com; 4 Chemistry Department, Federal University of Pernambuco, Recife 50740-560, Brazil; Email: petrus@pq.cnpq.br; 5 Nucleus of Research in Environmental Science, Catholic University of Pernambuco, Recife 50050-590, Brazil; Email: freitasdasilva@yahoo.com.br; 6 Nutrition Department, Federal University of Pernambuco, Recife 50670-901, Brazil; Email: tlmstamford@yahoo.com.br

**Keywords:** biopolymers, *Mucor circinelloides*, crystallographic properties, deacetylation, thermal analysis

## Abstract

Microbiological processes were used for chitin and chitosan production by *Mucor circinelloides* (UCP 050) grown in yam bean (*Pachyrhizus erosus* L. Urban) medium. The polysaccharides were extracted by alkali–acid treatment and structural investigations by X-ray diffraction, Fourier transform IR analysis, viscosity and thermal analysis by TG, DTG, and DTA were done. The highest biomass yield (20.7 g/L) was obtained at 96 hours. The highest levels of chitosan (64 mg/g) and chitin (500 mg/g) were produced at 48 and 72 hours, respectively. It was demonstrated that yam bean shows great potential as an economic medium and it is possible to achieve a good yield of chitosan with chemical properties that enable its use in biotechnological applications.

## 1. Introduction

Chitosan is a cationic, biodegradable, biocompatible, and bioactive amino polysaccharide, essentially composed of β-1,4-D-glucosamine (GlcNAc) linked to *N*-acetyl-D-glucosamine residues. This polymer has great economic value due to its versatile biological activities and chemical applications as an antimicrobial, antitumor, immuno-potentiator and wound healing agent, and in slow release drugs, water purification, dietary fiber, packaging films, coatings, among other uses [1,2,3,4,5,6].

Chitosan is a common constituent of fungal cell walls, particularly in the Zygomycetes class. It is produced by the chemical or spontaneous deacetylation of chitin, an insoluble linear β-1.4-*N*-acetyl-D-glucosamine (GlcNAc) polymer. Commercially, chitosan is obtained through the chemical deacetylation of crustacean chitin under strong alkali treatment [7,8,9,10]. Crustacean chitosan is inconsistent in its physical-chemical properties due to the variability in raw materials, the harshness of the isolation and conversion processes, the caustic effects of the chemicals used in the isolation process, and variability in the levels of deacetylation and protein contamination [11,12,13]. 

In order to obtain chitosan of a more consistent quality, filamentous fungi have been considered an attractive source for industrial applications because their specific products can be manufactured under standardized conditions [14,15,16,17,18,19]. This is achieved by careful manipulation of the growth parameters such as pH, and composition during the fermentation. These manipulations result in a varying molecular weight and degree of deacetylation of the chitosan, which influenced various properties, the application, and the biological response of the polymer [20,21,22]. 

The use of biomass from fungi has demonstrated great advantages, such as its independence from seasonal factors, ease of large-scale production, and the possible simultaneous extraction of chitin and chitosan. The extraction process is simple and cheap resulting in a reduction of the time and the cost required for production. In addition, absence of protein contamination in the co-polymers obtained from fungi biomass, especially those proteins that could cause allergic reactions in individuals with shellfish allergies [2,14,23,24,25]. It is worth highlighting that one interesting strategy to further reduction of the costs of fungi chitin and chitosan synthesis is the utilization of low cost alternative substrates [25].

Thus, the aim of this investigation was to produce chitin and chitosan by *Mucor circinelloides* (UCP 050) grown by submerse fermentation using the economic culture medium yam bean (*Pachyrhizus erosus* L. Urban), as substrate. The physicochemical characteristics and functional properties of the chitin and chitosan synthesized are also described. 

## 2. Results and Discussion

The growth profile of *M. circinellooides* (UCP 050) and chitin/chitosan production using yam bean medium is shown in Table 1.

**Table 1:** Profile of growth of *M. circinelloides* UCP 050 in yam bean medium at 28 °C, 150 rpm, during 96 hours of cultivation, describe glucose and protein consumption, pH ranger, biomass (g/L), chitin and chitosan yields (mg/mL).

*M. circinelloides/Cultivation time* (h)	Glucose consumption (g/L)	Nitrogen consumption (g/L)	pH	Biomass yield (g/L)	Chitin yield (mg/g)	Chitosan yield (mg/g)
0	11.4 ± 0.2	8.72 ± 0.09	7 ± 0.1	0.0 ± 0.0	260 ± 0.4	33 ± 0.1
24	9.6 ± 0.1	7.2 ± 0.2	5.9 ± 0.2	11.6 ± 0.6	320 ± 0.2	64 ± 0.2
48	3.95 ± 0.1	1.4 ± 0.09	4.8 ± 0.2	16.8 ± 0.4	500 ± 0.2	64 ± 0.2
72	3.16 ± 0.1	0.84 ± 0.1	4.5 ± 0.1	20.7 ± 0.5	500 ± 0.2	64 ± 0.4
96	2.08 ± 0.1	0.01 ± 0.07	4.2 ± 0.1	18.9 ± 0.5	500 ± 0.0	64 ± 0.1

Biomass production increased rapidly within 48 hours of growth, and reached a statistically significant (p = 0.003) higher production of biomass in 72 hours of cultivation, with average dry weight corresponding to 20.7 g/L (Table 1). These results are compared with the literature as verified in Table 2. 

**Table 2:** Chitin and chitosan production by *Mucor circinelloides* using yam bean as substrate.

Microorganisms	Substrate	Biomass (g.L^−1^)	Chitin (mg.g^−1^)	Chitosan (mg.g^−1^)	Reference
*Mucor circinelloides*	Yam bean	20.70	500	64	This study
*Cunninghamella* * elegans*	Yam bean	24.30	440	66	[25]
*Cunninghamella elegans*	Hesseltine and Anderson with added 5%NaCl and 6%glucose	24.40	388	70	[10]
*Cunninghamella bertholletiae*	Sugar cane juice	7.70	-	128	[14]
*Aspergillus niger*	Potato Dextrose Broth	9.00	-	107	[16]
*Lentinus edodes*	Potato Dextrose Broth	1.4	-	33	[16]
*Zygosaccharomyces rouxii*	Yeast Malt Extract Broth	4.4	-	36	[16]
*Candida albicans*	Yeast Malt Extract Broth	1.8	-	44	[16]

- Data not shown.

The *M. circinelloides* growth curve data for biomass, pH, nitrogen consumption, and glucose consumption is presented in Table 1. It demonstrated that the residual glucose and nitrogen were 2.08 g/L and 0.01 g/L, respectively. Similar results were reported by Andrade *et al.* [2] and Franco *et al.* [26]. Amorim *et al.* [14] suggested that the remaining glucose and nitrogen was due to nitrogenous compounds such as secondary metabolites present at the end of growth of the fungi metabolism and an excess of carbon source in the medium.

The pH of yam bean medium oscillates between 7 and 4, during the culture period (Table 1). The pH values decreased during the exponential phase, probably because of pyruvic acid formation caused by the high glucose and starch concentration in yam bean medium. Amorim *et al.* [27] and Stamford *et al.* [25] described constant pH values during the “lag” phase and the pH decreased during the exponential phase. This information on a higher amount of yam bean glucose and starch had been previously mentioned by Sarangbin and Watanapokasin [28], during citric acid production. Amorim *et al.* [27], Amorim *et al.* [14] reported that during the growth of *C. elegans* and *C. bertholletiae*, respectively, the pH of the media dropped during the first 24 hours and remained low (between 5 and 3) during the first 96 hours of cultivation, probably because of the interaction between the medium substrate and the release of ions from the cell. Table 1 shows the chitin and chitosan yield extracted, after each 24 hour period, from *M. circinelloides* (UCP 050), grown in yam bean medium during 96 hours of cultivation. The best yields of the polysaccharides (mg per gram of dry mycelia biomass) were obtained after 48 hours of culture for chitosan (64 mg/g or 6.4%) and after 72 hours for chitin (500 mg/g or 50%). These results are in agreement with Stamford *et al.* [25]. Similar results were reported by Tan *et al.* [17], who studied different Zygomycetes strains and observed that *Cunninghamella echinulata* was the best chitosan-producing strain, with a yield of approximately 7.0% of chitosan per mycelia dry weight. According to Chatterjee *et al.* [7], the production of chitosan from fungi was influenced by the composition of the growth medium, and the highest amount of chitosan from *Mucor rouxii* they reported was 7%, as shown in Table 2.

Chitosan production stabilized after 48 hours of culture, while chitin production increased up to 72 hours of culture, and decreased at 96 hours. The higher chitosan yields at 48 hours of growth suggest that during initial growth chitin is less crystalline and thus more susceptible to chitin deacetylase, and the chitosan formed by this enzyme prevails at acid pH (Table 1). According to Amorim *et al.* [1] the optimum pH for chitin deacetylase activity from Zygomycetes is pH 4.5. During *M. circinelloides* growth, the pH of the yam bean medium drops to 5-4 in the first 48 hours, and stabilizes at around 4 after 72 hours (Table 1), and shows a high metabolic interchange between the medium substrate uptake and the release of ions from the cells. Amorim *et al.* [27] reported that higher yields of chitosan were found within 24 hours of cultivation of *M. racemosus* and *C. elegans* at pH 3.5, which seems to also be a stimulating agent for the production of this biopolymer. 

The data in this study are in agreement with Nadarajah *et al.* [11], Pochanavanich and Suntornsuk [16], Amorim *et al.* [14], and Stamford *et al.* [25] who state that chitosan production by microorganisms is strongly dependent on the culture conditions, including cultivation time. Chung *et al.* [29] demonstrated that chitin and chitosan content in the cellular wall of fungi change according to the species and these polymers usually show higher values in Zygomycetes. 

The decline in the amount of extractable chitosan seen in the time-culture curve may be due to physiological changes in the cell wall of the fungi. Chitosan is produced in the fungal cell wall by deacetylation of its precursor, the nascent chitin. During the exponential phase, the ratio of free chitosan molecules is relatively high, due to the active growth. Once the culture enters the stationary growth phase, more of the chitosan is anchored to the cell wall of the Zygomycetes and binds to chitin and other polysaccharides so that extraction becomes more difficult [11,12,17].

**Figure 1:**
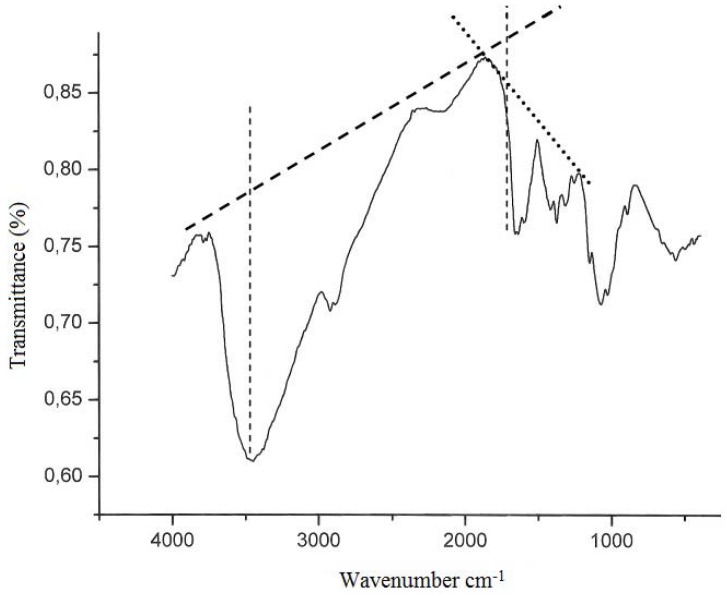
Infrared spectra of chitosan obtained from biomass of *M. circinelloides* UCP 050 and lignes of degree of acetylation (DD) determined according to Baxter *et al.* [6].

The characterization of chitin and chitosan obtained from *M. circinelloides* using yam bean medium by infrared spectroscopy (Figure 1) is similar to those reported in the literature [2,24,27]. The most significant features of the chitin and chitosan spectra are the amide bands at approximately 1665, 1555 and 1313 cm^−1^, which could be assigned to the C=O stretching, the N–H deformation in the CONH plane and the CN bond stretching plus CH_2 _wagging. In a similar way, chitin from *C. elegans* shows bands in the amide II region at 1153, 1378, and 1558 cm^−1^. The results are in agreement with Shigemasa *et al.* [30]. Andrade *et al.* [23], and Franco *et al.* [24], who reported that the chitin structure contains two types of amide group and both form C=O^….^N-H intermolecular bonds, but one is also an acceptor for the CH_2_OH group.

According to Santos *et al.* [8], deacetylation and the regeneration process cause disturbance in the initial crystalline reticulum of chitin, inducing a reordering of the hydrogen linking of chitosan. This can be observed in the central band at approximately 3483 cm^−1^ and 3305 cm^−1^, in the region of: (i) the axial deformation of OH, which appears as overlapping the band of axial deformation of NH indicating an intermolecular hydrogen linking formation; and at (ii) the displacement of the higher frequency band indicating an increase in the structural order. The data are in accordance with those reported in literature when comparing both chitin and chitosan infrared spectra obtained by microbiological methods [2,8,16,24,27].

Deacetylation degree (%DD) is an important parameter associated with the physical–chemical properties of chitosan, because it is linked directly to the chitosan cationic properties [16]. In the present study, chitosan obtained from *M. circinelloides* grown on yam bean medium presented 83% DD. That result is in accordance with Amorim *et al.* [27], Pochanavanich and Suntornsuk [16]; Chatterjee *et al.* [7], and Franco *et al.* [24], who all reported that the deacetylation degree of chitosan from fungi occurred between 80 to 90% DD.

The average viscosimetric molecular weight (*M_V_*) of chitosan from *M. circinelloides* obtained in this study is 2.70 × 10^4^ g/mol, which is considered relatively low [16]. The result is in agreement with the literature, which reports molar weights ranging between 1.0 × 10^4^ to 9.0 × 10^5^ g/mol [7,8,11]. Chitosan with a low molecular weight was reported to reduce the tensile strength and elongation of the chitosan membrane but to increase its permeability. Thus, fungal chitosan could have potential medical and agricultural applications [16].

**Figure 2:**
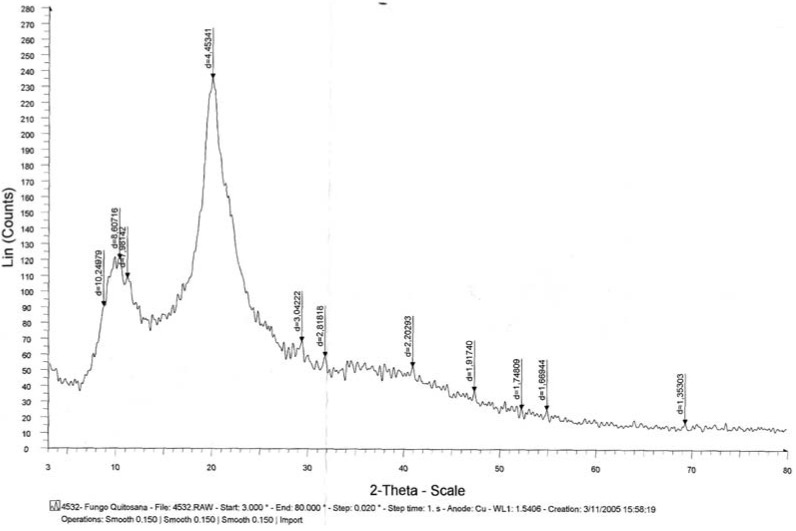
X-ray diffraction of chitosan obtained from *Mucor circinelloides* (UCP 050) biomass.

X-ray diffraction is commonly used to determine the polymorphic forms of a compound having different crystalline structures for which distinct powered X-ray diffraction patterns are obtained. These patterns are indicative of different spacing of the crystal planes, which provide strong evidence for polymorphic differences. In addition, it provides accurate measurements of the crystalline content, which greatly affects physical and biological properties of the polymer [7,15,31]. Figure 2 shows the powder diffraction pattern of chitosan from *M. circinelloides* grown in yam bean medium. Strong Bragg refractions were observed at an angle 20.0° 2*θ* (*d* = 4.4534 Å). From the above-mentioned result, it can be concluded that fungi chitosan displays an organized reticular structure. This is consistent with previous results from Chatterjee *et al.* [7]. In addition, Francis Suh [32] reported that chitosan crystallinity is related to the deacetylation degree function.

**Figure 3:**
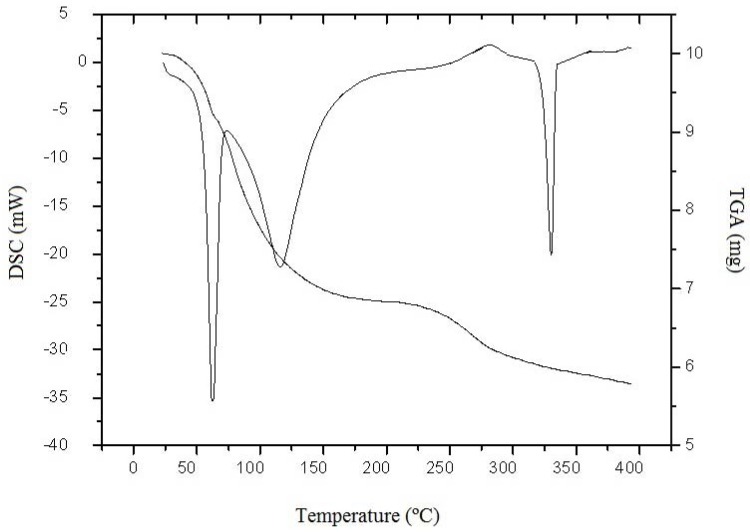
DSC and TGA termograms of chitosan from *Mucor circinelloides* UCP 050, under continuous flow of dry nitrogen gas (50 mL.min^−1^), at a heating rate of (10 °C min^−1^).

Polymers usually have a strong affinity for water and in the solid state these macromolecules may have a disordered structure that can be easily hydrated [33]. As is known, the hydration properties of chitosan depend on the primary and supramolecular structure [21]. Figure 3 shows the TGA and DSC curve of microbiological chitosan under N_2_ atmosphere between 25–400 °C. From the TGA curve it is observed that chitosan degraded in three stages. The first degradation stage can be explained as the loss of water. The second stage, which began at 170 °C, is due to the decomposition temperature of this polymer, with a carbonized residue formation, and the third weight loss point was at 335 °C which corresponds to the start of the consumption of the carbonized material. These results are in agreement with Liu *et al.* [34].

In the DSC curve, two peaks are observed (Figure 3). The first registered thermal event was a wide endothermic peak between 26 and 182 °C. According to Kittur *et al.* [21], the endothermic peak, related to the evaporation of water, is expected to reflect physical and molecular changes during N-deacetylation and carboxymethylation. The second thermal event registered, related to the polymer decomposition, was an endothermic peak. The endothermic effect of fungi chitosan is associated with the increased enthalpy, due to the presence of weak intramolecular hydrogen bonds [21]. This event is coherent with the TGA curve observations and agrees with the studies by Kittur *et al.* [21] and Santos *et al.* [8]. The latter suggested that there is an inverse relationship between thermal stability and deacetylation degree. However, no evidence was found for any influence of the molecular weight on thermal stability.

## 3. Experimental Section

### 3.1. Microorganism

*Mucor circinelloides* UCP 050 belongs to Culture Collection of Catholic University of Pernambuco, Recife, Brazil. It was originally isolated from mangrove sediment from Rio Formoso, Pernambuco, Brazil, and registered in the World Federation Culture for Collection-WFCC. The strain was maintained at 5 °C on Potato Dextrose Agar (PDA) slants.

### 3.2. Fermentation Medium Peparation

*M. circinelloides* was grown in yam bean Medium (*Pachyrhizus erosus* L. Urban) (chemical composition: total protein 8.72 g, starch 40.9 g, and glucose 11.14 g per liter of distilled water), and pH 7.0, for chitin and chitosan production. Tuberous roots of yam bean were provided by the Department of Agronomy of the Federal Rural University of Pernambuco, Recife, PE, Brazil. The surface of the tuberous roots was washed with water and soap to remove impurities. The yam bean medium was prepared from tuberous roots that had been peeled, round sliced (±1.5 cm), and boiled in distilled water in the ratio of 1:2 (w/v) for 45 minutes (after boiling began). The broth was cooled, filtered using Whatman n°1 filter paper and autoclaved at 121.5 °C for 15 minutes [25]. 

### 3.3. Culture Growth Conditions

The spores of *M. circinelloides* were harvested from cultures grown for seven days at 28 °C in Petri dishes containing PDA medium. A suspension was prepared and adjusted to 10^8^ spores/mL, using a hematocytometer for counting. For submerse cultivation of the fungus, spore suspension (10 mL, 10^8^ spores/mL) was inoculated in 1,000 mL Erlenmeyer flasks containing the fermentation medium (290 mL) and the flasks were incubated at 28 °C in an orbital shaker at 150 rpm, for 96 hours. The mycelia were harvested, washed twice in distilled and deionized water by filtration, utilizing a nylon membrane silkscreen (120 F), and were submitted to a lyophilization process. After lyophylization, the biomass was kept in a vacuum dissector until a constant weight was achieved. During the fermentation of *M. circinelloides* by submerse cultivation in yam bean medium, aliquots were collected every 24 hours in order to determine the biomass, pH, glucose and total nitrogen consumption [25]. 

### 3.4. Glucose and Nitrogen Consumption and pH Determination

Glucose consumption was determined by an enzymatic colorimetric method (Labtest^®^ Kit–Glucose oxidase). A standard curve was prepared using a range of glucose solutions (0.5 to 10.0 g/L). The Labtest^®^ Kit colorimetric method for proteins was used to determine nitrogen consumption, using a Spectronic Genesys 2 spectrophotometer. Determinations of pH were measured using a potentiometer (Digital Pontentiometer Quimis Mod. 400 A).

### 3.5. Chitin and Chitosan Extraction

The method used for chitin and chitosan extraction in this work followed the procedures described by Franco *et al.* [26]. Briefly, the process involved deproteination with 2% w/v sodium hydroxide solution (30:1 v/w, 90 °C, 2 h), and the separation of the alkali-insoluble fraction (AIF) by centrifugation (4,000 *g* 15 min.). The extraction of chitosan was carried out using the AIF fraction under reflux (10% v/v acetic acid 40:1 v/w, 60 °C, 6 h), and the separation of crude chitin by centrifugation (4,000 *g* 15 min.), and the precipitation of chitosan from the acid extract was by adjestment to pH 9.0 with 4 M NaOH solution. The crude chitin and chitosan obtained were washed on a coarse sintered-glass funnel with distilled water, ethanol, and acetone and air-dried at 20 °C to aconstant weight.

### 3.6. Chitin and Chitosan Characterization

#### 3.6.1. Infrared Spectroscopy (Deacetylation degree–DD%)

The degree of deacetylation for microbial chitin and chitosan were determined by means of infrared spectroscopy using the absorbance ratio A1655/A3450. A sample of fungal chitin and chitosan (2 mg) which had been dried overnight at 60 °C under reduced pressure, was thoroughly blended with 100 mg of KBr, to produce 0.5 mm thick disks. The disks were dried for 24 hours at 110 °C under reduced pressure. Infrared spectra were recorded with a Bruker 66 Spectrometer, using a 100 mg KBr disk for reference. The intensity of the maximum absorption bands was determined by the baseline method [25]. 

#### 3.6.2. Molecular Weight

The molecular weights of chitin and chitosan were determined by viscosimetry, using the procedure described by Santos *et al.* [8]. The viscosity of chitosan was determined using an AVS-350 viscometer (Schott-Geräte), type/capillary: Cannon-Fenske d_inside_ = 1.01 mm, at 25 °C. After obtaining the intrinsic viscosity from tables, K and a, were obtained for HAc/NaAc. K = 0.076, a = 0.76. The flow time was determined in seconds. Using the Mark-Houwinks equation, the average viscosimetric molecular weight was expressed in g/mol.

#### 3.6.3. Thermal Analysis

Thermogravimetric Analysis (TGA) and Differential Scanning Calorimetry (DSC) were carried out using Shimadzu model 50WS and Shimadzu model DSC-50WS thermal analysis instruments, respectively. An accurately weighed (10 mg) chitosan sample was placed in an aluminum cup and sealed. The experiment consisted of heating the samples from 0 to 400 °C under the continuous flow of dry nitrogen gas (50 mL.min^−1^), at a heating rate of 10 °C min^−1^.

#### 3.6.4. X-ray Diffraction

The X-ray diffraction patterns were determined using a wide-angle X-ray SIEMENS D5000 diffractometer and Ka, Cu radiation, with λ = 1.5406 A°. The voltage was 40 kV and the intensity 40 mA. The 2θ angle was scanned between 3° and 80°, and the count time was 1 second at each angle (0.02°) [7]. 

### 3.7. Statistical Analysis

The data were analyzed for significance using the Student’s t-test and chi-square test by means of the STATISTICA program version 6.0 of Statsolt Inc., USA. All experiments were carried out in triplicate and the results are expressed as mean ± S.D. 

## 4. Conclusions

The mycelium of *Mucor circinelloides* is a good source of chitin and chitosan. Yam bean (*Pachyrhizus erosus* L. Urban) showed great potential as an cheap substrate for medium production, and can provide a good yield of chitosan within two days of submerse fermentation with chemical properties that enable it to be used for biotechnological applications. Microbiological chitosan extraction process and can be optimized to improve the yield in large-scale production.
